# Correction: On the Use of Leaf Spectral Indices to Assess Water Status and Photosynthetic Limitations in *Olea europaea* L. during Water-Stress and Recovery

**DOI:** 10.1371/journal.pone.0118420

**Published:** 2015-03-06

**Authors:** The PLOS ONE Staff

## Notice of Republication

This article was republished on January 5, 2015, to correct errors in the text of the article and figures. Please download this article again to view the correct version. The originally published, uncorrected article and the republished, corrected article are provided here for reference. The publisher apologizes for the errors that were introduced during the typesetting process.

## Supporting Information

S1 File Originally published, uncorrected article. (PDF)

S2 File Republished corrected article. (PDF)
