# Supplementary material for: On the Use of Leaf Spectral Indices to Assess Water Status and Photosynthetic Limitations in Olea europaea L. during Water-Stress and Recovery
Source: PLoS One. 2014 Aug 19;9(8):e105165. doi: 10.1371/journal.pone.0105165 (PMC4138116; doi:10.1371/journal.pone.0105165)
Supplement: Appendix S1 — Summary of spectral measurements used within the study and key references. (DOC) [file pone.0105165.s001.doc]

Appendix 1: Summary of spectral measurements used within the study and key references. ^*^ λSWIR is the wavelength where the reflectance minimum at 1455 nm occurs in the SWIR region, and λNIR is the wavelength at 1272 nm, where a reflectance peak occurs in the NIR region.

| Index | Equation | Reference |
| --- | --- | --- |
| Photochemical reflectance index (PRI) | PRI = (*R*_531_ - *R*_570_) / (*R*_531_ + *R*_570_) | Gamon et al. 1992,  Gamon et al. 1997 |
| Water index (WI) | WI = *R*_900_ / *R*_970_ | Peñuelas and Filella 1998 |
| Relative depth index (RDI) | RDI = 100 ((*R*_max_ - *R*_min_) / *R*_max_) | Rollin and Milton 1998 |
| Water content reflectance index (WCRI) | WCRI = *R*_λSWIR_ / (*R*_λNIR_ - *R*_λSWIR_)^*^ | Sun et al. 2008 |
| Structural independent pigment index (SIPI) | SIPI = (*R*_800_-*R*_445_) / (*R*_800_-*R*_680_) | Peñuelas and Filella 1998 |
