# Supplementary material for: Correction: On the Use of Leaf Spectral Indices to Assess Water Status and Photosynthetic Limitations in Olea europaea L. during Water-Stress and Recovery
Source: PLoS One. 2015 Mar 6;10(3):e0118420. doi: 10.1371/journal.pone.0118420 (PMC4352039; doi:10.1371/journal.pone.0118420)
Supplement: S1 File — (PDF) [file pone.0118420.s001.pdf]

PLOS | ONE

On the Use of Leaf Spectral Indices to Assess Water Status and Photosynthetic Limitations in *Olea europaea* L. during Water-Stress and Recovery

Pengsen Sun[1], Said Wahbi[2], Tsonko Tsonev[3], Matthew Haworth[4], Shirong Liu[1], Mauro Centritto[4]*

1 Institute of Forest Ecology, Environment and Protection, Chinese Academy of Forestry, Beijing, P. R. China, 2 Laboratoire de Biotechnologie et Physiologie Végétale, Faculté des Sciences Semlalia, Université Cadi Ayyad, Marrakech, Morocco, 3 Institute of Plant Physiology and Genetics, Bulgarian Academy of Sciences, Sofia, Bulgaria, 4 Trees and Timber Institute, National Research Council, Sesto Fiorentino, Florence, Italy

Abstract

Diffusional limitations to photosynthesis, relative water content (RWC), pigment concentrations and their association with reflectance indices were studied in olive (*Olea europaea*) saplings subjected to water-stress and re-watering. RWC decreased sharply as drought progressed. Following rewatering, RWC gradually increased to pre-stress values. Photosynthesis (*A*), stomatal conductance (g_s), mesophyll conductance (g_m), total conductance (g_t), photochemical reflectance index (PRI), water index (WI) and relative depth index (RDI) closely followed RWC. In contrast, carotenoid concentration, the carotenoid to chlorophyll ratio, water content reflectance index (WCRI) and structural independent pigment index (SIPI) showed an opposite trend to that of RWC. Photosynthesis scaled linearly with leaf conductance to CO_2; however, *A* measured under non-photorespiratory conditions ($A_{1\%O2}$) was approximately two times greater than *A* measured at 21% [O_2], indicating that photorespiration likely increased in response to drought. $A_{1\%O2}$ also significantly correlated with leaf conductance parameters. These relationships were apparent in saturation type curves, indicating that under non-photorespiratory conditions, CO_2 conductance was not the major limitations to *A*. PRI was significant correlated with RWC. PRI was also very sensitive to pigment concentrations and photosynthesis, and significantly tracked all CO_2 conductance parameters. WI, RDI and WCRI were all significantly correlated with RWC, and most notably to leaf transpiration. Overall, PRI correlated more closely with carotenoid concentration than SIPI; whereas WI tracked leaf transpiration more effectively than RDI and WCRI. This study clearly demonstrates that PRI and WI can be used for the fast detection of physiological traits of olive trees subjected to water-stress.

Citation: Sun P, Wahbi S, Tsonev T, Haworth M, Liu S, et al. (2014) On the Use of Leaf Spectral Indices to Assess Water Status and Photosynthetic Limitations in *Olea europaea* L. during Water-Stress and Recovery. PLoS ONE 9(8): e105165. doi:10.1371/journal.pone.0105165

Editor: Pilar Hernandez, Institute for Sustainable Agriculture (IAS-CSIC), Spain

Received February 21, 2014; **Accepted** July 21, 2014; **Published** August 19, 2014

Copyright: © 2014 Sun et al. This is an open-access article distributed under the terms of the Creative Commons Attribution License, which permits unrestricted use, distribution, and reproduction in any medium, provided the original author and source are credited.

Funding: Financial support was provided by the National Natural Science Foundation of China (Grant No. 31290223), the Key Laboratory of Forest Ecology and Environment of State Forestry Administration of China, and the Ministero dellIstruzione dellUniversit della Ricerca of Italy: PRIN 2010-2011 PRO-ROOT and Progetto Premiale 2012 Aqua. The funders had no role in study design, data collection and analysis, decision to publish, or preparation of the manuscript.

Competing Interests: The authors have declared that no competing interests exist.

* Email: mauro.centritto@cnr.it

Introduction

The effective utilisation of renewable water resources in agriculture is a significant challenge in many areas of the globe. To mitigate the effects of increasing chronic water shortages, there is a need to develop and expand irrigation management practices. However, considering the unprecedented pressure on water resources for agriculture caused by rapidly growing water demand for urban and industrial uses, the expansion of irrigation may only become possible through the development of precision water saving irrigation techniques [1]. These are based on the real time detection of crop physiological status, and require the development of advanced, non-invasive phenotyping methods to monitor water relations and photosynthetic status in plants experiencing water-stress [2]. To this end, remotely sensed vegetation indices are increasingly being used as reliable cost-effective plant-based indicators to assess physiological traits associated with plant water status [3]. Furthermore, the real-time detection of plant physiological changes in regions subjected to drought is important for precision crop management and the estimation of terrestrial productivity. Therefore, improved knowledge of the relationship between leaf spectral and physiological responses under variable water conditions is of crucial importance.

Water deficit constrains all physiological processes involved in plant growth and development. These changes are part of a cascade of responses to drought that affect primary processes including tissue water relations and gas exchange mechanisms. It is well known that one of the earliest responses to water deficit is diminished stomatal conductance (g_s), because stomata act as control valves in the exchange of water vapor between leaf and the atmosphere to match soil water uptake rate with transpiration rate (*E*) to maintain plant water balance [4]. However, as g_s declines, CO_2 diffusion into the leaves also decreases, leading to reduced substomatal CO_2 concentration (C_i) [5]. This diffusional limitation to photosynthesis (*A*) in the gas phase is frequently associated with a coordinated change limiting CO_2 transport through the mesophyll (i.e., mesophyll conductance, g_m) [6,7]. As a consequence, during the early stages of drought stress, photosynthetic limitations have been shown to be predominantly caused by decreased total diffusive conductance (g_t, which is related to g_s and

g_m) leading to low chloroplastic CO_2 concentration (C_c) [2,7]. Alteration of photosynthetic metabolism generally becomes more prominent as water-stress progresses [5]. Nevertheless, there is still a degree of controversy on the relative importance of g_s, g_m or metabolic impairments in the limitation of A under drought (Lawlor and Cornic 2002) and during recovery upon re-watering [2].

Alterations in leaf water status, photosynthetic pigment concentrations and photosynthetic activity in turn lead to changes in spectral reflectance properties [3]. Reflectance in specific wavelength bands in the visible and near-infrared (NIR) region have potential applications in the estimation of plant water status [3]. Many spectral reflectance indices have been proposed in the direct monitoring of plant water-status; expressed as relative water content (RWC), leaf water potential and photosynthetic status. Among the water spectral indices, the NIR-based water index (WI) [3] is increasingly employed in monitoring water status; as recently demonstrated by Gutierrez et al., [8] to assess water relations in contrasting wheat genotypes, and by both Serrano et al., [9] and Marino et al., [10] to estimate vine water status at leaf and canopy levels. Furthermore, Sun et al., [11] developed a new index, the water content reflectance index (WCRI), which was inversely related to RWC. However, this relationship was only clear at low RWC values, suggesting that this water content-based spectral index may not be effective in detecting moderate stress.

The photochemical reflectance index (PRI) was originally developed to estimate rapid changes in the epoxidation state of xanthophyll pigments [12], a major component of non-photochemical quenching. However, PRI has been increasingly used to detect photosynthetic activity at different scales from plant biochemistry to the canopy level (see the review by Garbulsky et al., [13]. Dobrowsky et al., [14] found that heat and water-stress induced changes in steady-state chlorophyll fluorescence that were tracked by PRI; suggesting that PRI was a more effective real time indicator of photosynthetic function than indices based upon leaf water content and pigment concentration. Consistent with these findings, Ripullone et al., [15] observed strong relationships between photosynthetic activity and PRI in individual forest tree species subjected to water-stress. Similarly, significant linear relationships between PRI and A, and between PRI and g_s, were also demonstrated in *Solanum lycopersicum* [16] and *Quercus ilex* [17] subjected to water-stress, and at the tree canopy-level in irrigated and rainfed adult olive trees grown under field condition [10]. The PRI is not sensitive to sudden drought, but did track A, g_s and leaf water content during slow developing drought stress in *Olea europaea* [11,18]. However, to the best of our knowledge there is only one study that investigates the possibility of using PRI to track photosynthesis diffusional limitations (g_s and g_m) during the early stages of drought stress and during recovery upon re-watering [17].

In this study, diffusional limitations to photosynthesis, leaf water status, pigment concentrations and spectral reflectance indices were evaluated in olive (*Olea europaea* L.) plants during water-stress and recovery. The objective was to understand the links between simultaneous variations in photosynthetic diffusional limitations and PRI alongside leaf water relation parameters and WI by examining time course changes in response to water availability. Additionally, the constancy of other water content and pigment related spectral indices were also evaluated in response to water-stress and recovery.

Materials and Methods

Growing conditions and drought treatment

Three-year-old plants of olive (*Olea europaea* L., cv Leccino) were grown in 15 dm^3 pots filled with commercial soil in the National Research Council greenhouse in Montelibretti, Rome, Italy. Temperature within the greenhouse was maintained at 25–27°C, photosynthetically active radiation followed the natural light regime and relative humidity ranged between 60–70%. Large volume 15 dm^3 pots relative to the *Olea europaea* plants were chosen to avoid any potential effects of root restriction generating root to shoot signals that may interfere with the response of the plants to drought [19,20]. Furthermore, the g_s and PRI values of well-watered and drought treated plants were indistinguishable in the early stages of drought treatment and the values recorded in the drought treatment plants subsequently returned to pre-stress levels following re-watering in the recovery period, indicating that potential root restriction did not influence the effect of drought on simultaneous measurement of reflectance and gas exchange under conditions of water-deficit. The plants were regularly watered to pot water capacity and fertilized with Hoagland solution once a week to supply mineral nutrients at free access rates [21,22] for the first two months after the onset of the growing season. On the afternoon preceding the initiation of the experiment (Day 0), plants were fully irrigated and allowed to drain the excess water over-night. Then half of the plants were water-stressed by withholding water until the seedlings showed symptoms of severe water-stress, while the remaining half of the seedlings continued to be well-watered to pot capacity. On the afternoon of day 18 water-stressed plants were irrigated with 500 cm^3 of water as supplementary irrigation. On the evening of the Day 23, the water-stressed seedlings were then re-watered daily to pot capacity over a 7-day recovery period. Drought stress treatment was performed during May - June. The plants were fully randomized and five replicate plants per treatment were analyzed for all measurements.

Gas exchange and fluorescence measurements

Simultaneous measurement of gas exchange and fluorescence were conducted on five leaves per plant (these values were then averaged to produce a mean value for the individual plant, and then the mean of five replicate plants taken for a given treatment value) using a LI-6400-40 portable infrared gas-analyser (Li-Cor, Lincoln, NE, USA). All gas exchange measurements were made between 11.00 and 15.00 h, with the leaf chamber set to a saturating photosynthetic photon flux density (PPFD) (1300 μmol m^{-2} s^{-1}), relative humidity of the air ranging between 45–55% and at a leaf temperature of 25°C. To reduce diffusion leaks through the chamber gasket [23], a supplementary external chamber gasket composed of the same polymer foam was added to create an interspace between the two gaskets (i.e. a double-gasket design with a 5 mm space separating the internal and external gaskets). Then the CO_2 and H_2O gradients between the in-chamber air and pre-chamber air were minimized by feeding the IRGA exhaust air into the interspace between the chamber and the pre-chamber gaskets [24]. Instantaneous measurement of steady-state photosynthesis (A), transpiration (E), stomatal conductance (g_s), and $\Delta F/F_m'$ (i.e. the quantum yield of PSII in the light) were made on five plants per treatment after removal of stomatal limitation of A by lowering the external atmospheric [CO_2] (C_a) to 50 ppm as described by Centritto et al., [6]. Measurements of dark respiration (R_d) were performed at ambient [CO_2] concentration in the dark on the same leaves by switching off the light in the leaf cuvette; when CO_2 release from the leaf had become stable for approximately five to ten minutes this was

recorded and considered to represent R_d. Mesophyll conductance to CO_2 diffusion (g_m), the inverse of the total resistance encountered by CO_2 across the leaf mesophyll, was calculated using the variable J method. One percent oxygen for the measurement of A under non-photorespiratory conditions was produced using a Brooks Instruments mass flow controllers (Brooks Instruments, Hatfield, PA, USA) and a gas mixing system connected to cylinders of compressed pure nitrogen and oxygen gas, this was then connected to the gas input line of the LiCor Li6400. Then g_m in olive leaves was calculated following Aganchich et al., [30], whereas total diffusion conductance (g_t) was calculated as: $g_t = g_s\, g_m/(g_s + g_m)$.

Determination of relative water content and pigment concentrations

Leaf samples for relative water content (RWC) and pigment concentrations were taken immediately after the gas exchange and spectral measurements. Fully-developed leaves were detached and weighed to determine leaf fresh mass (F_M). After measuring leaf area, using a leaf area meter (LI3100, LI-COR Inc., Lincoln, NE, USA), leaves were then covered with a plastic bag and allowed to rehydrate with the cut-end under water in a dark cold room at 5°C for18 h. Following rehydration, each leaf was weighed to determine saturated mass (S_M), and then oven-dried at 80°C for 48 hours to determine dry mass (D_M). RWC was finally calculated as follows: $RWC = (F_M - D_M)/(S_M - D_M)$.

The carotenoid and total chlorophyll (*Chl*, a and b) concentration was measured in intact leaf tissues by immersion in N,N-dimethylformamide (DMF). Leaf discs with a total area of 1.5 cm^2 were placed into glass vials containing 2 cm^3 DMF and immediately placed in darkness at 4°C in an orbital shaker set to 100 rpm for 4 h. The absorbance (A_B) of the solution was then read on a spectrophotometer (Perkin Elmer, Norwalk, CT) at 663.8, 646.8, and 480 nm, using DMF as a blank. The pigment concentrations were calculated according to the following equations [25]:

$$\text{Total chlorophyll } (Chl_a + Chl_b) = (12A_{B663.8} - 3.11A_{B646.8}) + (20.78A_{B646.8} - 4.88A_{B663.8})$$

$$\text{Carotenoid} = (1000A_{B480} - 1.12Chl_a - 34.7Chl_b)/245.$$

Spectral measurements and indices

Spectral measurements were carried out in the laboratory, by a portable spectroradiometer (Field spec FR 350–2500 nm, ASD Inc., Boulder, CO, USA). As the reflectance properties of leaves alter with age [26], all of the leaves analysed were of approximately the same age, less than one year-old from the uppermost part of the canopy. The instrument measures spectral reflectance between 350 and 2500 nm to a resolution of 3 nm. The instrument automatically calculates the reflectance value as the ratio between the incident radiation reflected from the surface target and the incident radiation reflected by a reference spectral panel. The reference spectral panel material can be regarded as a Lambertian reflector. The Field spec FR may be operated with different lenses that control the field of view, in this study an 8° lens was used to restrict the field of view of the instrument. Reflectance spectra (R) were collected from a distance of 5.0 cm from the sample, which was fixed on a dark platform. To reduce instrument noise each spectral signature was calculated from the average of 100 scans. A further check of the stability of the reflected signal was performed by taking the white reflectance at the beginning and end of each sample. Reflectance spectra were preprocessed using the View Spec Pro (version 5.6, ASD, Inc.) software. A summary of the spectral measurements used within the study are presented in Appendix S1.

Statistics

Data were tested using a simple factorial ANOVA (two-way maximum interactions) and Tukey *post-hoc* test. The statistical analysis was performed by Sigma Plot 11.0 (Systat software, Inc., San Jose, CA, USA). Linear and non-linear curve-fitting (Sigma Plot 11.0), which minimizes the difference between observed and predicted values, were used for regression analyses.

Ethics Statement

No permits were required for the experimental analysis of *Olea europaea* plants that were bought commercially and complied with all relevant regulations and did not involve an endangered or protected species.

Results

Figure 1 shows the time-course of RWC, PRI, A, g_s, g_m and g_t during the study period of soil drying and subsequent recovery. As expected, relative water content (Fig. 1a) was strongly affected by the water-regime ($P<0.001$), decreasing first slowly (Days 1–8) and then sharply (Days 10–16) as drought progressed. RWC was significantly increased ($P<0.05$) by supplementary irrigation (Day 20) and then following re-watering to pot water capacity (Days 23–30). The whole experimental process resulted in a "W-shaped" time course in RWC. In fact, upon relief of the water-stress, RWC increased very rapidly to pre-stress levels. The response of PRI to the drought and recovery cycle (Fig. 1b) closely followed the pattern of RWC.

Photosynthesis (Fig. 1c), g_s, g_m, and g_t (Fig. 1d) were also strongly affected ($P<0.001$) by water availability. Water-stressed saplings exhibited a significant decrease in A even during the early phase of the drying cycle (Fig. 1c). When water-stress became very severe (Day 16), A measured in ambient air was almost completely inhibited and only slightly, but not significantly, increased following the supplementary irrigation (Day 20). At low [O_2], A measured under non-photorespiratory conditions ($A_{1\%O2}$), was increased by ~75% in well-watered plants at the beginning and at the end of the experiment. In comparison to control values of $A_{1\%O2}$, significant decreases in $A_{1\%O2}$ were only observed in severely stressed plants. It is interesting to note that the ratio between $A_{1\%O2}$ and A increased during water-stress, reaching a value of about two on Day 20 following the supplementary irrigation, and a similar value one day after re-watering on Day 24. Mild water-stress also induced significant decreases in g_s (Fig. 1d); in a similar pattern to A, g_s approached zero when soil water decreased to a level where there was no longer water available within the pot to sustain transpiration, and then did not significantly respond to supplementary irrigation. Upon relief of water-stress, g_s and A increased rapidly to pre-stress levels; but their recovery was less rapid than that of RWC. Interestingly, seven days after re-watering, A and g_s significantly exceeded pre-stress values by about 25% and 31%, respectively. Mesophyll conductance to CO_2 diffusion and, in turn, g_t mirrored the trend in g_s during the drought and recovery cycle (Fig 1d).

Stomatal and mesophyll conductance values were not only similar in magnitude but also linearly correlated ($P<0.001$)

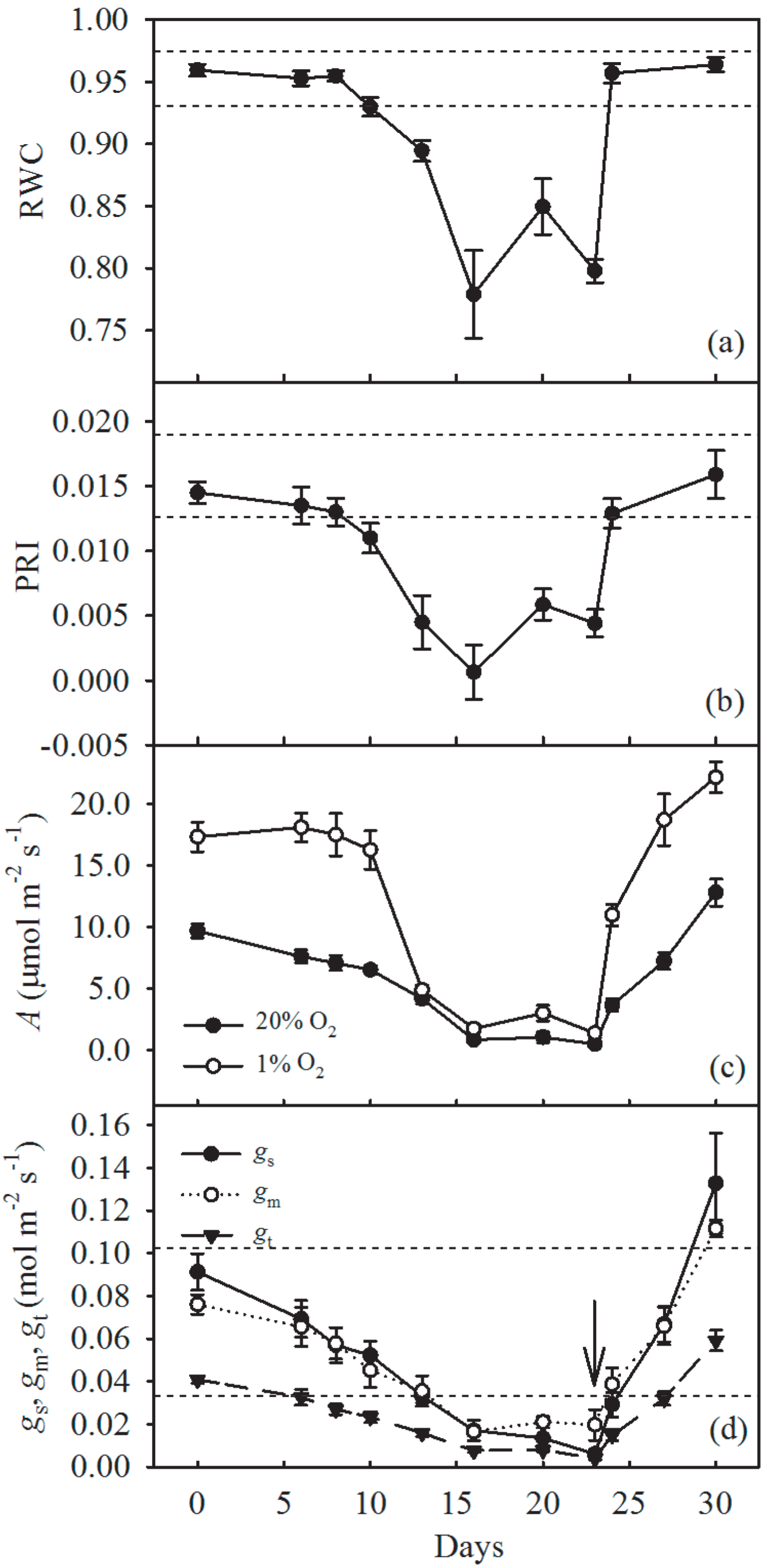

1.00
0.95
0.90
0.85
0.80
0.75
RWC
(a)
0.020
0.015
0.010
0.005
0.000
-0.005
PRI
(b)
20.0
15.0
10.0
5.0
0.0
A (μmol m-2 s-1)
20% O2
1% O2
(c)
0.16
0.14
0.12
0.10
0.08
0.06
0.04
0.02
0.00
gs, gm, gt (mol m-2 s-1)
gs
gm
gt
(d)
0
5
10
15
20
25
30
Days

Figure 1. Time courses of leaf (a) relative water content (RWC), dashed horizontal lines indicate the range of values (mean ± one standard deviation) recorded in the well-watered plants over the duration of the experiment; (b) photochemical reflectance index (PRI) (dashed horizontal lines as in 1a); (c) photosynthesis (*A*) (measured in both ambient air and in air with 1% [O_2] (*A*1%O2), and; (d) stomatal conductance (g_s), mesophyll conductance (g_m) and total conductance (g_t) of olive saplings grown during and after the drought cycle (days 1–23), dashed horizontal lines indicate the range of g_s values recorded in the well-watered plants. ↑ = end of the drying cycle. Data points are means of five plants (five leaves per plant) ±1 SEM.
doi:10.1371/journal.pone.0105165.g001

(Fig. 2). There was a significant linear correlation between A and g_s ($r^2 = 0.966$, $P<0.001$), when measurements conducted on control and water-stressed leaves were pooled (Fig. 3a). Similar linear relationships were also found between A and g_m ($r^2 = 0.959$, $P<0.001$) (Fig. 3b) and A and g_t ($r^2 = 0.969$, P<0.001) (Fig. 3c). Furthermore, $A_{1\%O2}$ scaled significantly with leaf conductance parameters (Fig. 3d,e,f). In contrast to A, the responses of $A_{1\%O2}$ to g_s ($r^2 = 0.945$, $P<0.001$), g_m ($r^2 = 0.965$, $P<0.001$), and g_t ($r^2 = 0.955$, $P<0.001$) took the form of a saturating curve.

After pooling together of all measurements during the drought and recovery cycle, there were significant exponential relationships between the gas-exchange parameters A (Fig. 4a), g_s, g_m and g_t (Fig. 4b), when plotted against RWC. The pre-stress and recovery (i.e., high RWC) gas-exchange parameters showed higher variation than those recorded during water-stress (Fig.4a,b). Stomatal conductance increased more rapidly than g_m and g_t during the late water-stress relief period (i.e., at leaf RWC ranging from 0.9 to 0.96) (Fig. 1d, Fig. 4b). The relationship between PRI and RWC was also non-linear ($r^2 = 0.935$, $P<0.001$), with RWC varying from 0.77 to 0.96 during the study period (Fig. 4c). The PRI showed less variability than the gas exchange parameters as RWC recovered to pre-stress levels. The PRI was also significantly correlated with to A (Fig. 4d), g_m (Fig. 4e) and g_t (Fig. 4e).

The water content-related reflectance indices, RDI (Fig. 5a) and WI (Fig 5c), showed a similar time-course response to that of RWC (Fig. 1a); they decreased significantly as water-stress progressed and significantly increased following the relief of water-stress. In contrast, WCRI (Fig. 5b) increased significantly as water-stress became severe, and recovered quickly and completely upon re-watering to pot water capacity. The supplementary irrigation (Day 20) altered RDI significantly ($P<0.05$), but had a less apparent effect on both WI and WCRI. Overall, RDI (Fig. 5d), WCRI (Fig. 5e) and WI (Fig. 5f) showed significant linear relationships with RWC. However, WCRI differed from the other two indices by being inversely related to RWC. The RDI and WCRI were found to exhibit comparatively stronger correlations with RWC than WI.

The RDI showed significant non-linear relationships with g_s (Fig. 6a) and E (Fig. 6b); whereas, WI scaled linearly with g_s (Fig. 6c) and E (Fig. 6d). In contrast, there was an inverse curvilinear relationship between WCRI and both g_s (Fig. 6e) and E (Fig. 6f). It is noteworthy that RDI and WCRI were highly sensitive to both g_s and E under severe stress conditions ($g_s <$ 0.06 mol m^{-2} s^{-1}), whereas this sensitivity decreased sharply in mild-stress or optimal conditions ($g_s > 0.06$ mol m^{-2} s^{-1}).

Carotenoid concentration (Fig. 7a) was significantly affected ($P<0.05$) by water-stress. Carotenoid concentration increased as water-stress progressed; resulting in a 34% increase when water-stress was most severe (Days 16 and 23), and then decreased significantly following both the supplementary irrigation (Day 20) and re-watering to pot water capacity (Day 24). Chlorophyll concentration was inversely associated to that of carotenoids (data not shown), and this led to a progressive decrease in the carotenoid to chlorophyll ratio as water-stress progressed, and to a correspondingly rapid increase in response to the supplementary irrigation and relief of water-stress (Fig. 7b). The time-course of structural independent pigment index (SIPI, i.e. a measure of carotenoid to chlorophyll *a* ratio) (Fig. 7c) mirrored that of the overall carotenoid to chlorophyll ratio. Finally, significant linear relationships to carotenoid concentration were found between both PRI (Fig. 8a) and SIPI (Fig. 8b).

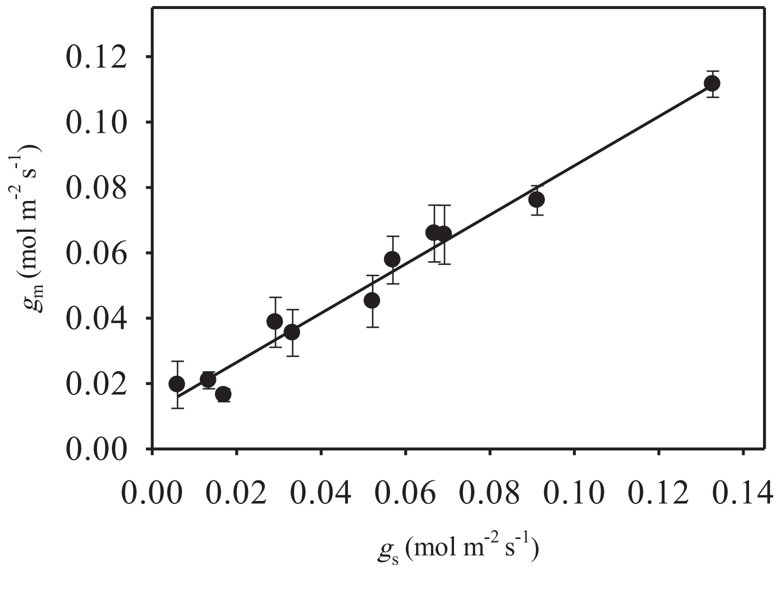

Figure 2. Linear relationships between mesophyll conductance (g_m) and stomatal conductance (g_s) ($r^2 = 0.978$, $P<0.001$) in olive saplings grown during and after the drought cycle.
doi:10.1371/journal.pone.0105165.g002

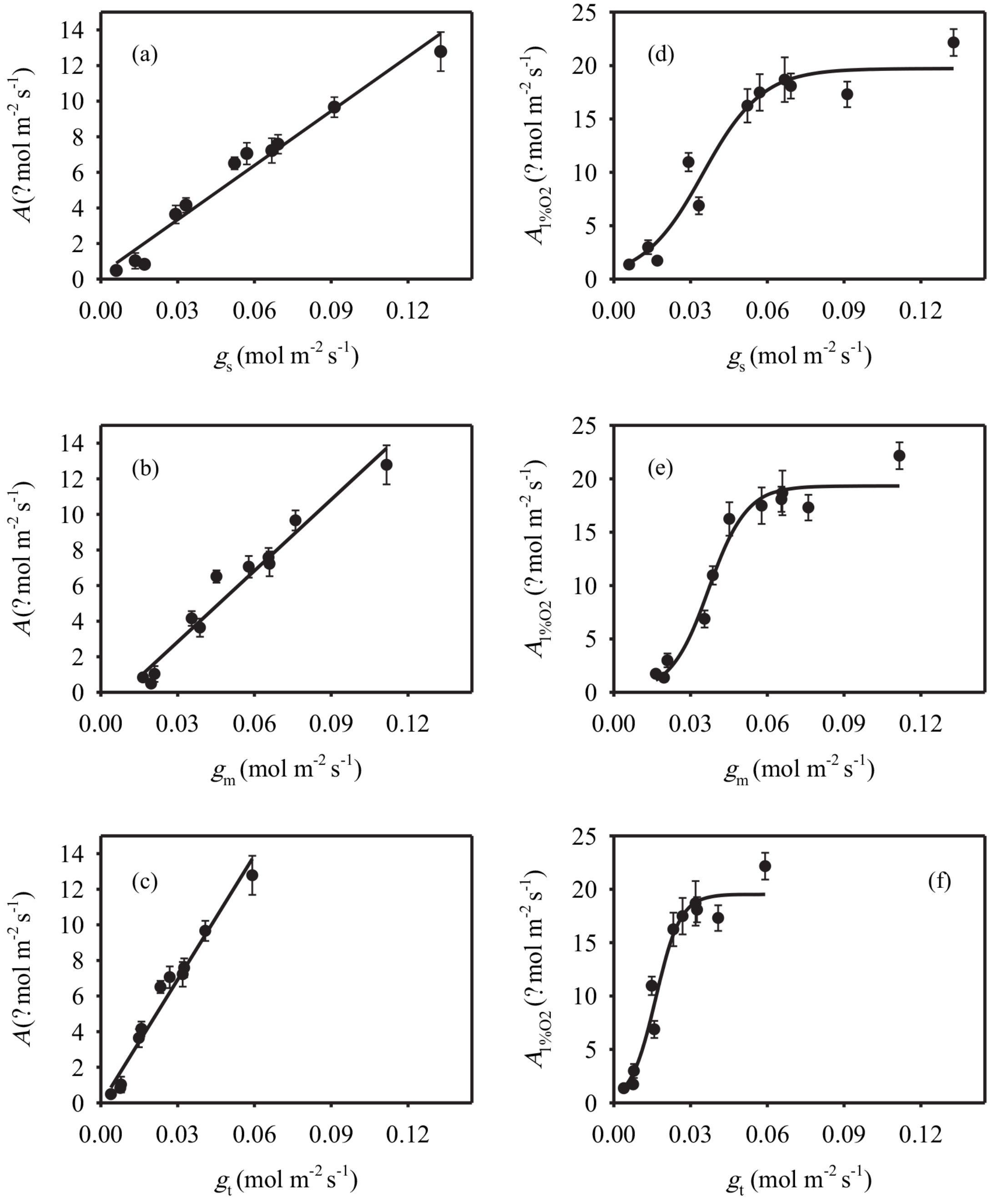

Figure 3. Relationships of photosynthesis (*A*), measured in ambient air (a, b, c) and in air with 1% [O_2] (*A*1%O2) (d, e, f), and (a, d) stomatal conductance (g_s), (b, e) mesophyll conductance (g_m), and (c, f) total conductance (g_t) in olive saplings grown during and after the drought cycle.
doi:10.1371/journal.pone.0105165.g003

Discussion

Water shortage is a crucial problem limiting plant productivity across large areas of the globe. Changes in biogeophysical cycles (that is increased evaporative demand and water holding capacity of the air) associated with global warming are expected to further worsen the water crisis in arid and semi-arid zones, and to also increasingly affect temperate regions [27]. To mitigate the effects of drought on plant growth it essential to improve our knowledge of plant functional responses to soil drying. In this study, we assessed a cascade of changes in leaf water relations, pigment concentrations, photosynthetic CO_2 diffusional limitations in olive saplings subjected to water-stress and recovery by using spectral reflectance indices to monitor the water-status and photosynthetic function of olive trees.

The time-course in leaf water status, as expressed by RWC (Fig. 1a) and PRI (Fig. 1b), in response to water availability was mirrored by that of the photosynthetic parameters. Furthermore, as observed in other studies [2], the decline of both *A* (Fig. 4a) and leaf conductance parameters (Fig. 4b) was significantly correlated to RWC. We observed a gradual decrease of *A* (Fig. 1c) in water-stressed saplings during the early period of the drought treatment

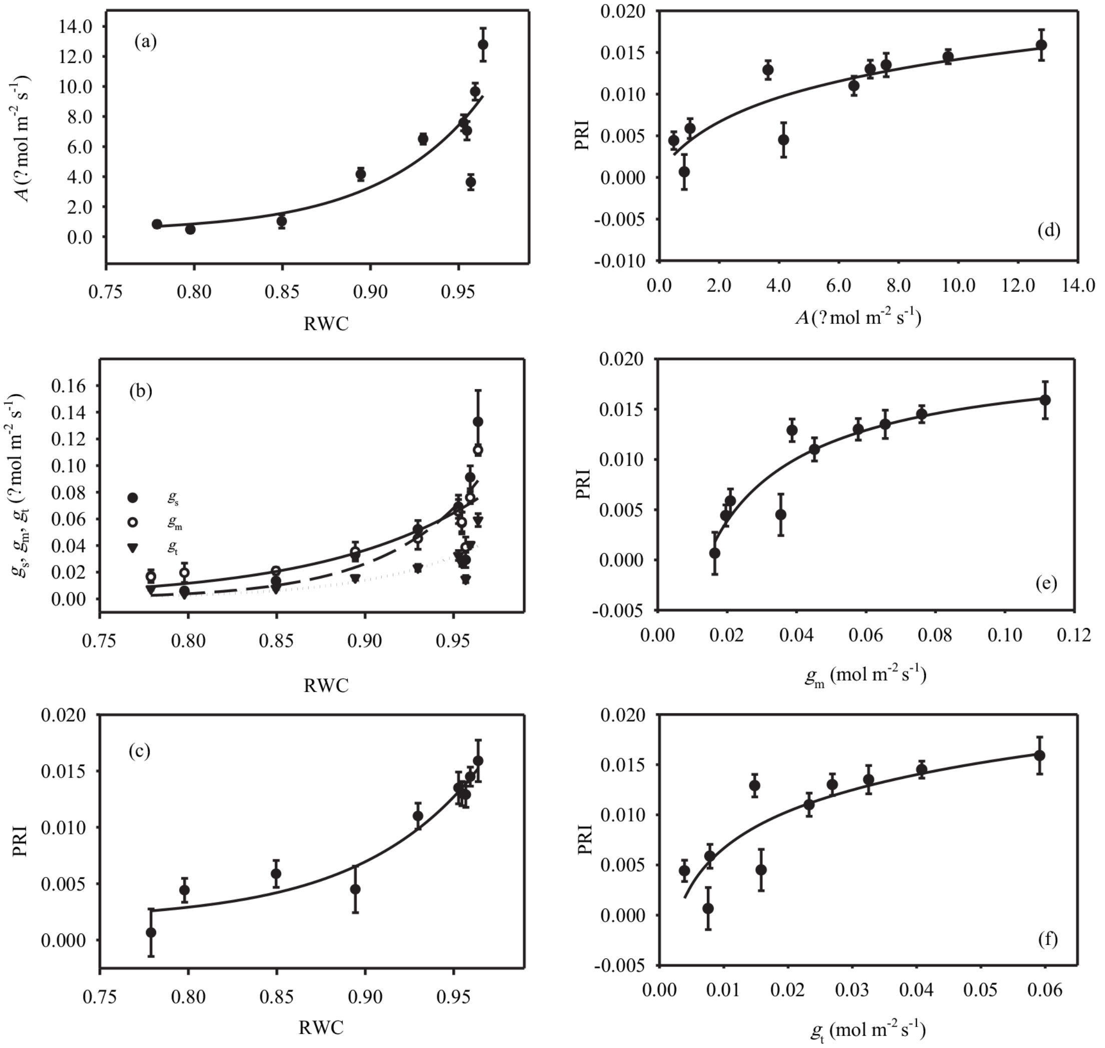

Figure 4. Relationships between relative water content (RWC) and (a) photosynthesis (*A*) ($r^2 = 0.742$, $P = 0.008$), (b) stomatal conductance (g_s, ——) ($r^2 = 0.667$, $P = 0.005$), mesophyll conductance (g_m, ----) ($r^2 = 0.748$, $P = 0.003$) and total conductance (g_t, ·····) ($r^2 = 0.653$, $P < 0.02$), and (c) photochemical reflectance index (PRI) ($r^2 = 0.935$, $P < 0.001$), and between PRI and (d) *A* ($r^2 = 0.762$, $P = 0.001$, (e) g_m ($r^2 = 0.844$, $P < 0.001$), and (f) g_t ($r^2 = 0.725$, $P = 0.002$) in olive saplings grown during and after the drought cycle.
doi:10.1371/journal.pone.0105165.g004

(Day 10). The reduction in conductance values of CO_2 diffusion (g_s, g_m and g_t) (Fig. 1d) followed similar patterns, suggesting that during the early phase of the drought stress *A* was limited by reduced C_c [5,28,29]. There are numerous studies suggesting that under water-stress, *A* is mostly limited by reduced C_i due to stomatal closure [5]. However, information about the contribution of g_m during water-stress is still lacking [28,29]; although new evidence indicates that g_m is linearly correlated to g_s (Fig. 2) [2,6], and an important component in the determination of water use efficiency [7]. When water-stress reached severe levels (Day 16), *A* was almost fully inhibited, and upon relief of the water-stress *A* gradually recovered to the pre-stress level in parallel with g_s, g_m and g_t. The *A* and g_s values observed in this study, in both well-watered and stressed conditions, are consistent with the findings of previous work performed on young pot-grown olive plants [11,30] and mature olive trees grown under field conditions [10,31,32,33]. Moriana et al., [31] and Marino et al., [10] observed that some degree of metabolic limitation to *A* occurred in olive trees exposed to severe summer drought. However, the rapid increase in *A* and its complete restoration to pre-stress levels after one week of stress-relief, in addition to the linear relationships between *A* and leaf conductance parameters (Fig. 3a,b,c), may indicate that *A* was likely mainly inhibited by impaired CO_2 uptake [34].

Photosynthesis measured under non-photorespiratory conditions ($A_{1\%O2}$) changed in parallel with *A* (Fig. 1c). Assuming that the difference between $A_{1\%O2}$ and *A* is an approximate measure for photorespiration, it is evident that this parameter was stimulated dramatically at the onset of water stress. This is may have been caused by reductions in C_c as a result of decreases in g_t (Fig. 1d). Reduced C_c may then lead to increased oxygenation of ribulose-1,5-bisphosphate (RuBP) by Rubisco, thus increasing photorespiration, not only relative to photosynthesis, but also in absolute terms [35,36]. For example, photorespiration in four grape varieties increased under moderate water-stress and helped maintain a relatively high photochemical efficiency of PSII and rapid recovery of *A* after re-watering [37]. However, as photorespiration depends directly on RuBP recycling in the Benson-Calvin cycle, which in turns depends on C_c, severe drought may also result in reduced rates of photorespiration [35]. This may explain the progressive reduction of $A_{1\%O2}$ under

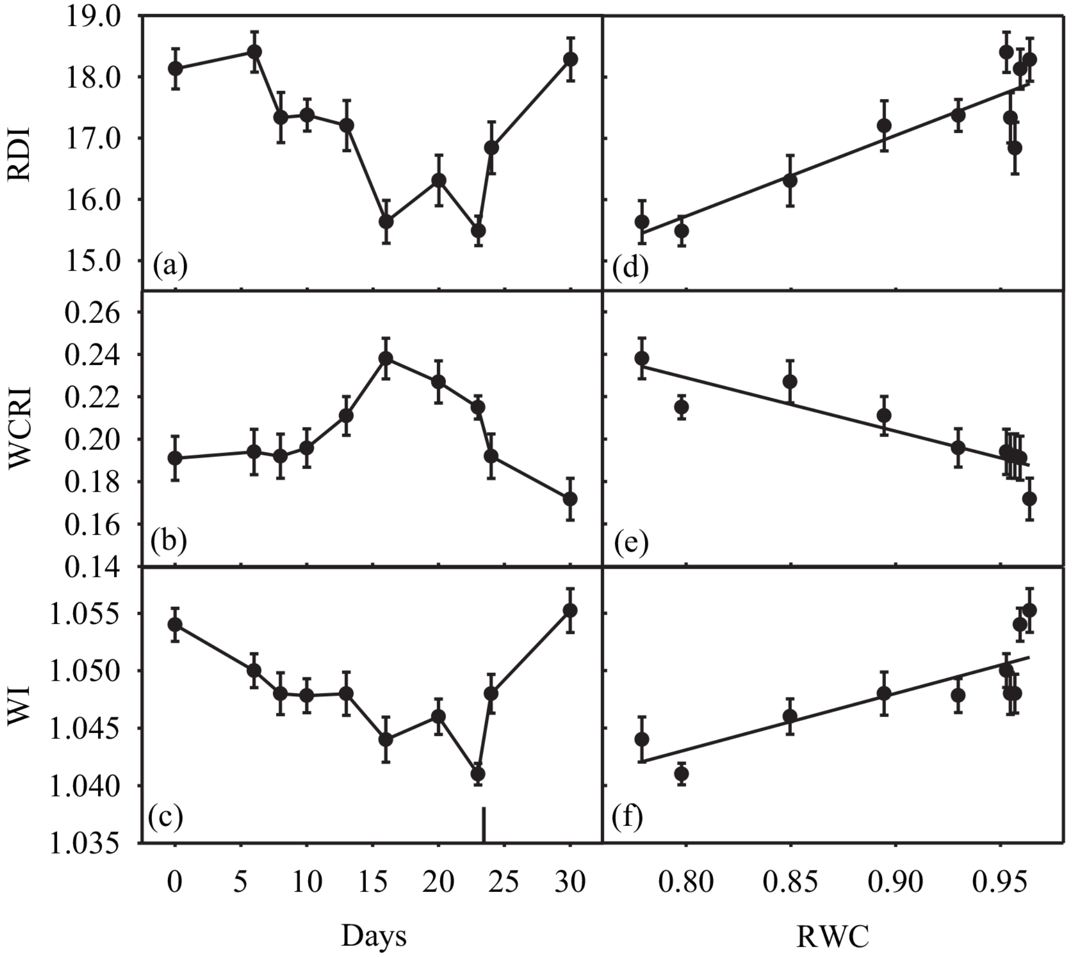

Figure 5. Time courses of (a) relative depth index (RDI), (b) water correlated reflectance index (WCRI) and (c) water index (WI), and relationships between relative water content (RWC) and (d) RDI (r^2 = 0.803, *P*<0.001), (e) WCRI (r^2 = 0.814, *P*<0.001) and (f) WI (r^2 = 0.675, *P* = 0.004) in olive saplings grown during and after the drought cycle (days 1–23). ↑ = end of the drying cycle. Data points are means of five plants (five leaves per plant) per measurement ±1 SEM.
doi:10.1371/journal.pone.0105165.g005

conditions of severe water-stress. Conversely, the increase in $A_{1\%O2}$ observed after the supplementary irrigation and relief of water-stress (Days 20 and 24, respectively), when $A_{1\%O2}$ was approximately two-fold higher than A, may likely be attributed to the corresponding increase in g_m and C_c. Furthermore, the saturating responses of $A_{1\%O2}$ to g_m (Fig. 3d), g_s (Fig. 3e), and g_t (Fig. 3f) imply that under non-photorespiratory conditions, conductance of CO_2 is not the major limitation to A. Thus, because broad-leaved sclerophyll plants, such as olive, possess inherently low leaf conductance, they may have a competitive advantage over mesophyllous vegetation in the future as atmospheric [CO_2] rises [38].

As CO_2 transport from the ambient air to the active site of Rubisco decreases in response to progressive water-stress, A and the quantum yield of photosystem II (PSII) become progressively inhibited. In turn, the proportion of the PPFD energy absorbed by the photosynthetic antenna and dissipated as heat increases, while that used for photochemistry declines. This photo-protective reaction is associated with the de-epoxidation state of xanthophylls [39]. PRI is based on a normalized difference of the 531 and 570 nm bands where xanthophyll pigments absorption occurs and, thus can be used as a robust proxy of radiation use efficiency [12] and photosynthetic activity [13]. The PRI, was dramatically affected by water availability (Fig. 1b), and exhibited close correlation to RWC (Fig. 4c) and A (Fig. 4d). The strong relationship between PRI and RWC (P<0.001) observed in this study is in keeping with previous studies showing that PRI is a good indicator of leaf water status [15]. Furthermore, PRI detected at tree canopy level, has been recently found to be correlated to A in field-grown mature olive plants under rainfed and well-watered conditions [10]. Significant correlations between PRI and A have also been found in *Arbutus unedo* [15], *Ceratonia siliqua* [40], *Solanum lycopersicum* [16] and *Quercus ilex* [15,17] subjected to water-stress in different environmental conditions. Photosynthesis is mainly limited by diffusive limitations to CO_2 uptake [7,28,29], unless water-stress becomes particularly severe to the point where there is virtually no water available to support transpiration [4,34]; consequently, the direct detection of diffusional limitations by spectral indices may be of great importance in the detection of plant function under stress conditions. In this study, significant relationships between g_t (Fig. 4f) and g_m (Fig. 4e) were established. Similar results were also observed in a study of *Q. ilex* [17] response to water-stress and recovery. These relationships, which were similar to that observed between A and PRI (Fig. 4d), can be viewed as supportive indications of the suitability of PRI in tracking photosynthetic activity.

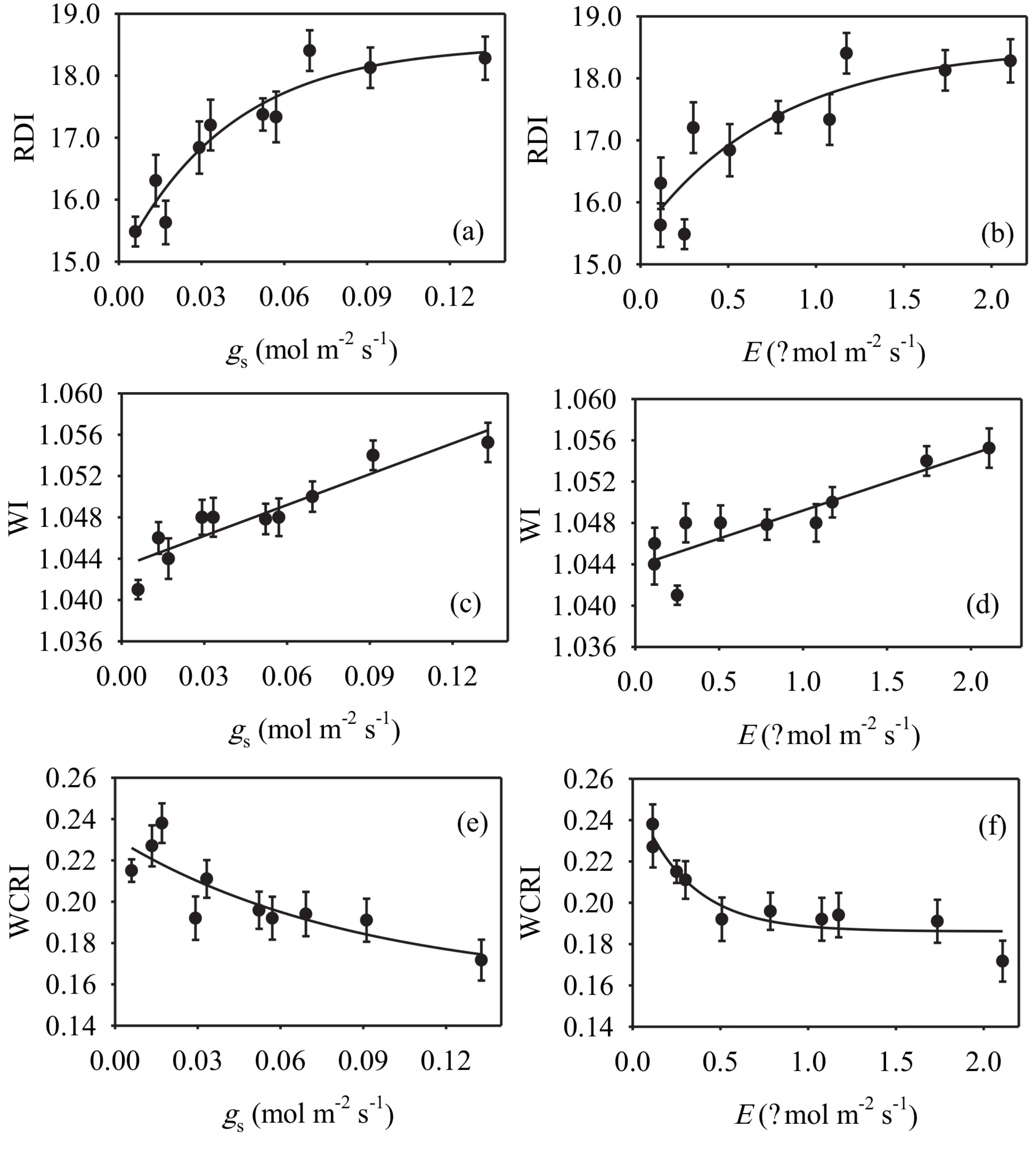

Figure 6. Relationships between relative depth index (RDI) and (a) leaf stomatal conductance (g_s) ($r^2 = 0.908$, $P < 0.001$) and (b) leaf transpiration (E) ($r^2 = 0.796$, $P < 0.01$), between WI and (c) g_s ($r^2 = 0.863$, $P < 0.001$) and (d) E ($r^2 = 0.801$, $P < 0.001$), and between WCRI and (e) g_s ($r^2 = 0.717$, $P < 0.012$) and (f) E ($r^2 = 0.902$, $P < 0.002$) in olive saplings grown during and after the drought cycle (days 1–23).
doi:10.1371/journal.pone.0105165.g006

Variation in PRI can be affected by short-term changes in xanthophyll epoxidation state and over longer time-scales by shifts in the size of carotenoid and chlorophyll pools [41,42,43]. Similarly, time-course variations in SIPI, a pigment-based reflectance index [3], in response to water status (Fig. 7c) may reflect corresponding changes in the ratio of carotenoids to chlorophyll. In this study, carotenoid concentration (Fig. 7a) and the carotenoid to chlorophyll ratio (Fig. 7b) were responsive to water availability, as they significantly increased as the water-stress progressed and then decreased following supplementary irrigation and re-watering to pot water capacity. This may indicate the ability of olive trees to up- and down-regulate photo-protective thermal dissipation mechanisms by xanthophyll cycle carotenoids in response to water status [39]. The PRI (Fig. 8a) and SIPI (Fig. 8b) both inversely reflected changes in carotenoid concentrations. Interestingly, SIPI was less effective than PRI in gauging carotenoid concentration. Unlike PRI, which is estimated in the green-yellow region of the radiation spectrum (i.e., within the

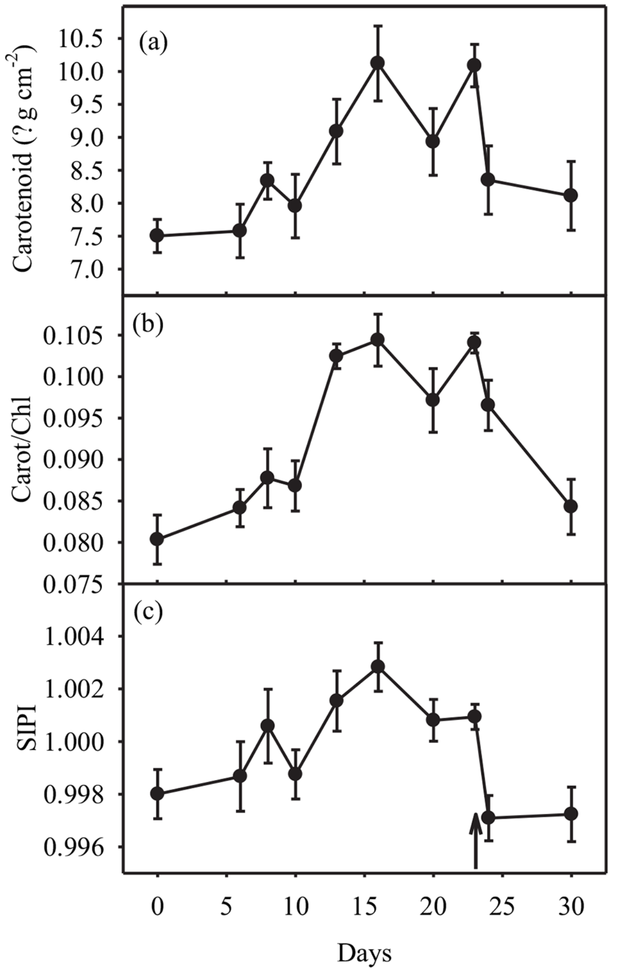

Figure 7. Time courses of (a) leaf carotenoid concentration (μg cm^{-2}), (b) carotenoid to chlorophyll ratio (Carot/Chl), and (c) structural independent pigment index (SIPI) in olive saplings grown during and after the drought cycle (days 1–23). ↑ = end of the drying cycle. Data points are means of five plants (five leaves per plant) per measurement ±1 SEM.
doi:10.1371/journal.pone.0105165.g007

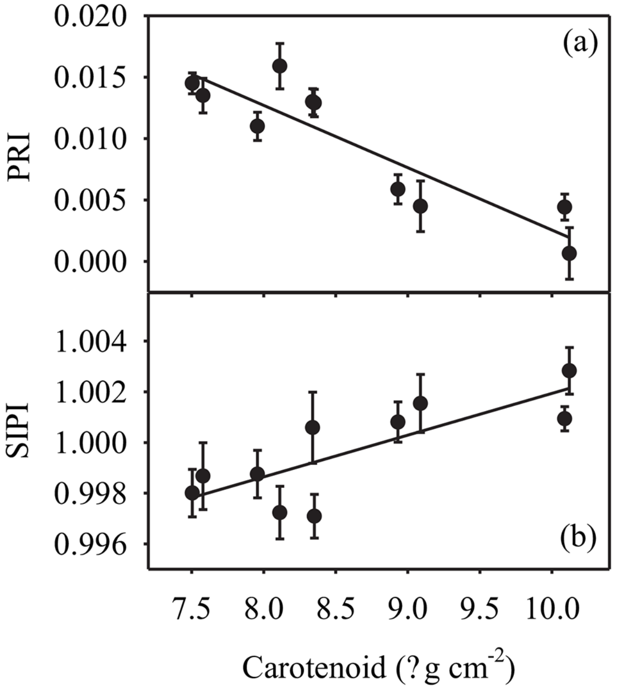

Figure 8. Linear relationships between carotenoid concentration (μg cm^{-2}) and (a) photochemical reflectance index (PRI) ($r^2 = 0.813$, $P < 0.001$) and (b) structural independent pigment index (SIPI) ($r^2 = 0.622$, $P < 0.007$) in olive saplings grown during and after the drought cycle.
doi:10.1371/journal.pone.0105165.g008

carotenoid absorption spectra), SIPI is estimated using blue and red bands that are sensitive to both carotenoid and chlorophyll pigment changes. Therefore, the combined variations in both carotenoid and chlorophyll pools may have contributed to the higher variability of SIPI in comparison to that of PRI. However, taken together, these results reinforce the growing body of literature showing that PRI [10,13,41], and to a lesser extent SIPI, [10,44] are correlated to photosynthetic parameters.

The three water-related reflectance indices, RDI, WI and WCRI, were significantly affected during the water-stress cycle and re-watering. The RDI (Fig. 5a) and WI (Fig. 5c) mirrored the time course of RWC (Fig.1a); whereas WCRI (Fig. 5b) increased as water-stress progressed and decreased upon rewatering. These differential responses resulted in significant direct positive relationships between RWC and both RDI (Fig. 5d) and WI (Fig. 5f), and a significant negative relationship between RWC and WCRI (Fig. 5e). As all these indices are formulated by several water absorption wavelengths, these linear relationships indicate the consistent sensitivity of RDI, WI and WCRI to mild or severe drought conditions. Furthermore, these three spectral indices were also significantly correlated to g_s and E (Fig. 6), due to the close dependence of stomata behavior and transpiration upon leaf water status. The RDI (Fig. 6a,b) and WCRI (Fig. 6e,f) had curvilinear relationships with g_s and E, and were consequently more sensitive as E and g_s decreased, and less sensitive under mild-stress and optimal conditions. Conversely, WI was linearly related to both g_s (Fig. 6c) and E (Fig. 6d), and as a consequence was more sensitive to these two physiological parameters than RDI and WCRI. This differential sensitivity may result from the different wavelengths used in calculating RDI, WI and WCRI, as middle infrared wavelengths are used to estimate RDI and WCRI while near-infrared wavelengths are used to calculated WI. It is noteworthy that Marino et al., [10] demonstrated in a recent study of mature olive trees that WI, estimated at tree canopy level, was the most accurate predictive index of plant water status and whole-plant transpiration. Similarly, Serrano et al., [9] found that WI was a good indicator of vineyard water status. We therefore suggest that WI is the most reliable tool in predicting parameters of plant water-status.

Conclusion

Drought is a complex syndrome affecting several leaf biophysical and biochemical properties that subsequently influence leaf reflectance spectra. In the present study, we examined the reliability of water absorption and pigment based spectral indices to assess changes in leaf water status, pigment concentration and photosynthetic traits during water-stress and recovery. In general, PRI was very sensitive to leaf water status, pigments concentrations and A. It is noteworthy that PRI also significantly tracked leaf CO_2 conductance. These relationships may reflect the tight control that diffusional limitations exert on A under water-stress and then following relief from water-stress. The WI was the most

reliable index in the prediction of plant water-status. Finally, *A* was likely mainly inhibited by leaf CO_2 conductance parameters during water-stress; whereas under non-photorespiratory conditions, CO_2 conductance was not the major limitation to photosynthesis. In consideration of a predicted future elevated atmospheric [CO_2], it is possible that sclerophyll plants will likely out-perform mesophyllous vegetation.

Supporting Information

Appendix S1 Summary of spectral measurements used within the study and key references.
(DOC)

Acknowledgments

The comments of two anonymous reviewers significantly improved this manuscript. The authors are grateful to the Scientific Cooperation Agreements between CNR and the Chinese Academy of Forestry; CNR and the Centre National pour la Recherche Scientifique et Technique of Morocco.

Author Contributions

Conceived and designed the experiments: PS SW TT SL MC. Performed the experiments: PS SW TT. Analyzed the data: MC MH. Contributed reagents/materials/analysis tools: MC TT. Wrote the paper: MC MH.

References

1. Fereres E, Orgaz F, Gonzalez-Dugo V (2011) Reflections on food security under water scarcity. Journal of Experimental Botany 62: 4079–4086
2. Centritto M, Lauteri M, Monteverdi MC, Serraj R (2009) Leaf gas exchange, carbon isotope discrimination, and grain yield in contrasting rice genotypes subjected to water deficits during the reproductive stage. Journal of Experimental Botany 60: 2325–2339
3. Peñuelas J, Filella L (1998) Visible and near-infrared reflectance techniques for diagnosing plant physiological status. Trends in Plant Science 3, 151–156
4. Centritto M, Brilli F, Fodale R, Loreto F (2011a) Different sensitivity of isoprene emission, respiration, and photosynthesis to high growth temperature coupled with drought stress in black poplar (*Populus nigra*). Tree Physiology 31: 275–286
5. Lawlor DW, Cornic G (2002) Photosynthetic carbon assimilation and associated metabolism in relation to water deficits in higher plants. Plant, Cell and Environment 25: 275–294
6. Centritto M, Loreto F, Chartzoulakis K (2003) The use of low [CO_2] to estimate diffusional and non-diffusional limitations of photosynthetic capacity of salt-stressed olive saplings. Plant Cell and Environment 26: 585–594
7. Flexas J, Niinemets U, Gallé A, Barbour MM, Centritto M, et al. (2013) Diffusional conductances to CO_2 as a target for increasing photosynthesis and photosynthetic water-use efficiency. Photosynthesis Research, 117: 45–59
8. Gutierrez M, Reynolds MP, Klatt AR (2010) Association of water spectral indices with plant and soil water relations in contrasting wheat genotypes. Journal of Experimental Botany 61: 3291–3303
9. Serrano L, González-Flor C, Gorchs G (2010) Assessing vineyard water status using the reflectance based water index. Agriculture, Ecosystems and Environment 139: 490–499
10. Marino G, Pallozzi E, Cocozza C, Tognetti R, Giovannelli A, et al. (2014) Assessing gas exchange, sap flow and water relations using tree canopy spectral reflectance indices in irrigated and rainfed *Olea europaea* L. Environmental and Experimental Botany 99: 43–52
11. Sun P, Grignetti A, Liu S, Casacchia R, Salvatori R, et al. (2008) Associated changes in physiological parameters and spectral reflectance indices in olive (*Olea europaea* L.) leaves in response to different levels of water stress. International Journal of Remote Sensing 29: 1725–1743
12. Gamon JA, Peñuelas J, Field CB (1992) A narrow-waveband spectral index that tracks diurnal changes in photosynthetic efficiency. Remote Sensing of Environment 41: 35–44
13. Garbulsky MF, Peñuelas J, Gamon JA, Inoue Y, Filella I (2011) The photochemical reflectance index (PRI) and the remote sensing of leaf, canopy and ecosystem radiation use efficiencies: a review and meta-analysis. Remote Sensing of Environment 115: 281–297
14. Dobrowski SZ, Pushnik JC, Zarco-Tejada PJ, Ustin SL (2005) Simple reflectance indices track heat and water stress-induced changes in steady-state chlorophyll fluorescence at the canopy scale. Remote Sensing of Environment 97: 403–414
15. Ripullone F, Rivelli AR, Baraldi R, Guarini R, Guerrieri R, et al. (2011) Effectiveness of the photochemical reflectance index to track photosynthetic activity over a range of forest tree species and plant water statuses. Functional Plant Biology 38: 177–186
16. Sarlikioti V, Driever SM, Marcelis LFM (2010) Photochemical reflectance index as a mean of monitoring early water stress. Annals of Applied Biology 157: 81–89
17. Tsonev T, Wahbi S, Sun P, Sorrentino G, Centritto M (2014) Gas exchange, water relations and their relationships with photochemical reflectance index in *Quercus ilex* plants during water stress and recovery. International Journal of Agriculture and Biology 16: 335–341
18. Suárez L, Zarco-Tejada PJ, Sepulcre-Cantó G, Pérez-Priego O, Miller JR, et al. (2008) Assessing canopy PRI for water stress detection with diurnal airborne imagery. Remote Sensing of Environment 112: 560–575
19. Arp WJ (1991) Effects of source-sink relations on photosynthetic acclimation to elevated CO_2. Plant, Cell & Environment 14: 869–875
20. Ismail A, Hall A, Bray E (1994) Drought and pot size effects on transpiration efficiency and carbon isotope discrimination of cowpea accessions and hybrids. Functional Plant Biology 21: 23–35.
21. Magnani F, Centritto M, Grace J (1996) Measurement of apoplasmic and cell-to-cell components of root hydraulic conductance by a pressure-clamp technique. Planta 199: 296–306
22. Centritto M, Lee HSJ, Jarvis PG (1999) Long-term effects of elevated carbon dioxide concentration and provenance on four clones of Sitka spruce (*Picea sitchensis*) I. Plant growth, allocation and ontogeny. Tree Physiology 19: 799–806
23. Flexas J, Díaz-Espejo A, Berry J, Cifre J, Galmés J, et al. (2007) Analysis of leakage in IRGA's leaf chambers of open gas exchange systems: quantification and its effects in photosynthesis parameterization. Journal of Experimental Botany 58: 1533–1543
24. Rodeghiero M, Niinemets U, Cescatti A (2007) Major diffusion leaks of clamp-on leaf cuvettes still unaccounted: how erroneous are the estimates of Farquhar, et al. model parameters? Plant, Cell and Environment 30: 1006–1022
25. Wellburn AR (1994) The spectral determination of Chlorophylls *a* and *b*, as well as total carotenoids, using various solvents with spectrophotometers of different resolution. Journal of Plant Physiology 144: 307–313
26. Peñuelas J, Filella I, Gamon JA (1995) Assessment of photosynthetic radiation-use efficiency with spectral reflectance. New Phytologist 131: 291–296
27. Dai A (2010) Drought under global warming: a review. WIREs Climate Change 2: 45–65
28. Flexas J, Ribas-Carbó M, Diaz-Espejo A, Galmés J, Medrano H (2008) Mesophyll conductance to CO_2: current knowledge and future prospects. Plant, Cell and Environment 31: 602–621
29. Loreto F, Centritto M (2008) Leaf carbon assimilation in a water-limited world. Plant Biosystems 142: 154–161
30. Aganchich B, Wahbi S, Loreto F, Centritto M (2009) Partial root zone drying: regulation of photosynthetic limitations and antioxidant enzymatic activities in young olive (*Olea europaea*) saplings. Tree Physiology 29: 685–696
31. Moriana A, Villalobos FJ, Fereres E (2002) Stomatal and photosynthetic responses of olive (*Olea europaea* L.) leaves to water deficits. Plant, Cell and Environment 25: 395–405
32. Centritto M, Wahbi S, Serraj R, Chaves MM (2005) Effects of partial rootzone drying (PRD) on adult olive tree (*Olea europaea*) in field conditions under arid climate. II. Photosynthetic responses. Agriculture, Ecosystems and Environment 106: 303–311
33. Diaz-Espejo A, Nicolas E, Fernández JE (2007) Seasonal evolution of diffusional limitations and photosynthetic capacity in olive under drought. Plant, Cell and Environment 30: 922–933
34. Brilli F, Tsonev T, Mahmood T, Velikova V, Loreto F, et al. (2013) Ultradian variation of isoprene emission, photosynthesis, mesophyll conductance and optimum temperature sensitivity for isoprene emission in water-stressed *Eucalyptus citriodora* saplings. Journal of Experimental Botany 64: 519–528
35. Wingler A, Quick WP, Bungard RA, Bailey KJ, Lea PJ, et al. (1999) The role of photorespiration during drought stress: an analysis utilizing barley mutants with reduced activities of photorespiratory enzymes. Plant, Cell and Environment 22: 361–373
36. Sage RF (2013) Photorespiratory compensation: a driver for biological diversity. Plant Biology 15: 624–638
37. Guan XQ, Zhao SJ, Li DQ, Shu HR (2004) Photoprotective function of photorespiration in several grapevine cultivars under drought stress. Photosynthetica 42: 31–36
38. Centritto M, Tognetti R, Leitgeb E, Střelcová K, Cohen S (2011). Above ground processes - Anticipating climate change influences. In: Bredemeier M, Cohen S, Godbold DL, Lode E, Pichler V, Schleppi P, eds. Forest Management and the Water Cycle: An Ecosystem-Based Approach. Ecological Studies 212, Springer, pp 31–64
39. Demmig-Adams B, Adams WWIII (2000) Harvesting sunlight safely. Nature 403: 371–374

40. Osório J, Osório ML, Romano A (2012) Reflectance indices as nondestructive indicators of the physiological status of *Ceratonia siliqua* seedlings under varying moisture and temperature regimes. Functional Plant Biology, 39: 588–597
41. Sims DA, Gamon JA (2002) Relationships between leaf pigment content and spectral reflectance across a wide range of species, leaf structures and developmental stages. Remote Sensing of Environment 81: 337–354
42. Guo J, Trotter CM (2004) Estimating photosynthetic light-use efficiency using the photochemical reflectance index: variations among species. Functional Plant Biology 31: 255–265
43. Filella I, Porcar-Castell A, Munne-Bosch S, Back J, Garbulsky MF, et al. (2009) PRI assessment of long-term changes in carotenoids/chlorophyllratio and short-term changes in de-epoxidation state of the xanthophyllcycle. International Journal of Remote Sensing 30: 4443–4455
44. Ollinger SV (2011) Sources of variability in canopy reflectance and the convergent properties of plants. New Phytologist 189: 375–394.
